# Employee reactions to CSR in the pursuit of meaningful work: A case study of the healthcare industry

**DOI:** 10.3389/fpsyg.2022.969839

**Published:** 2022-11-14

**Authors:** Josine L. Janssen, Evgenia I. Lysova, Christopher Wickert, Svetlana N. Khapova

**Affiliations:** Department of Management and Organisation, Vrije Universiteit Amsterdam, Amsterdam, Netherlands

**Keywords:** meaningful work, meaningfulness, healthcare, micro-CSR, corporate social responsibility (CSR)

## Abstract

With the growing interest in the microfoundations of corporate social responsibility (‘micro-CSR'), many questions linger regarding how the workforce reacts to CSR, which has consequences for their meaningful work experiences. To address this lack of understanding, we conducted an inductive, comparative case study of two healthcare organisations to examine how employees experience meaningful work through reacting to their organisation's CSR initiatives. We demonstrate how CSR triggers employees' meaning-making of work, which takes the form of a misalignment perceived between CSR at the strategic-level and CSR as it is implemented at the employee-level, limiting the experiences of meaningful work. We identify four proactive behaviours in which employees engage to infuse their work with meaning as a way of dealing with this experienced misalignment. We consolidate these behaviours into a typology of meaning-infusing behaviours in the context of CSR. Specifically, we found that when guided by the need for making a positive impact on their beneficiaries, employees engage in what we call ‘reshaping work for impact' next to ‘collectively enabling impact'. In contrast, when guided by the need for having a sense of meaningful membership, employees are guided by either ‘creating a sense of belonging' or ‘envisioning prosocial potential'. Through these behaviours, they either navigate within given organisational structures or enact new ones. Overall, we expand research on the CSR–meaningful work relationship, emphasising the role of employees' proactive behaviours in understanding their experiences and reactions to CSR initiatives in their pursuit of meaningful work. Moreover, we highlight implications for micro-CSR research and practice.

## Introduction

Researchers have called for more studies on the microfoundations of CSR, often labelled as ‘micro-CSR' (Aguinis and Glavas, 2012; Wickert and de Bakker, 2018; Gond and Moser, 2021; Girschik et al., 2022), to better understand how CSR manifests itself in organisations, and what the role of employees in this process is. Given our focus on the role of employees, we define CSR as the way employees (including managers) “within an organisation think about and discuss relationships with stakeholders as well as their roles in relation to the common good, along with their behavioural disposition with respect to the fulfilment and achievement of these roles and relationships” (Basu and Palazzo, 2008, p. 124). Understanding how employees evaluate, drive, and react to CSR is significant as they are the key agents within organisations that continue to implement CSR strategies; however, how the workforce experiences CSR remains a critical but undertheorized topic (Gond et al., 2017).

Employee-focused micro-level CSR research – a relatively nascent but rapidly growing field (Gond et al., 2017; Gond and Moser, 2021) – posits that one crucial way CSR can affect employees is through their subjective experience of work. Specifically, scholars have focused on the topic of work meaningfulness in the context of CSR (e.g., Pratt et al., 2013; Aguinis and Glavas, 2019; Opoku-Dakwa and Rupp, 2019) and it has been suggested as an important avenue for future research (Jones, 2019). Meaningful work is broadly defined as a perception of individuals that their work is purposeful and significant (Rosso et al., 2010). Considering the increasing importance of work meaningfulness, moral standards, and sustainability to the workforce (Lysova et al., 2019) as well as to organisations (Dhingra et al., 2021), it is timely and theoretically valuable to study meaningful work and its relationship with CSR. Ethicists have further pointed out that meaningful work is a basic moral need of employees (Yeoman, 2014), and it is considered a moral responsibility of organisations to enable it (Michaelson et al., 2014).

Yet, research that addresses the CSR–meaningful work relationship has been mainly conceptual (e.g., Michaelson et al., 2014; Glavas, 2016; Aguinis and Glavas, 2019; Opoku-Dakwa and Rupp, 2019), with some studies quantitatively testing this relationship (e.g., Glavas and Kelley, 2014; Raub and Blunschi, 2014; Brieger et al., 2020). A notable exception is a study by Seivwright and Unsworth (2016), which inductively analysed how employees make sense of CSR and argued that CSR is perceived as embedded (not peripheral) when it provides a sense of doing meaningful work. How exactly employees find meaningful work in the context of CSR, however, remains a critical gap in the literature. With the present study, we respond to calls for more qualitative research in micro-CSR (Glavas, 2016; Gond et al., 2017) and calls to further integrate the concepts of CSR and meaningful work (Aguinis and Glavas, 2019; Gond and Moser, 2021) as we explore how employees experience and react to CSR initiatives in their pursuit of meaningful work.

We conducted an inductive, comparative case study of two healthcare organisations that made their CSR profile explicit through actions and communication (e.g., through their mission statement). Studying this sector became particularly insightful in light of the COVID-19 pandemic and attention needs to be given to the working conditions of frontline healthcare workers in CSR research (Crane and Matten, 2021). Moreover, examining how CSR affects meaningful work is timely as healthcare organisations are struggling with understaffing, and meaningful work being an important predictor of employee well-being and withdrawal intentions (Allan et al., 2019). Similarly, Russo (2016) suggested that the social responsibility of healthcare organisations centres above all on the social and ethical impact on patients and employees (Russo, 2016). Considering that for these employees, ‘making a difference' is a core source of meaning and purpose in their work (Colby et al., 2001), we expect the reactions of employees to organisational CSR initiatives in their pursuit of meaningful work to be particularly salient in this context, and therefore insightful for deriving theoretical conclusions.

In this study, we illuminate how employees proactively infuse their work with meaning when they perceive a misalignment between CSR at the strategic level and CSR as it is implemented in their work – a finding that emerged from our data. We found that they do so by either shaping the boundaries of their job and work environment or by cognitively shaping their work experience, ultimately aimed at enhancing the meaningfulness of their work. We consolidate these behaviours into a typology of four types of meaning-infusing behaviours in the context of CSR. Specifically, we found that when guided by the need for making a positive impact on their beneficiaries, employees engage in what we call ‘reshaping work for impact' next to ‘collective enabling of impact'. In turn, when guided by the need for having a sense of meaningful membership, they are guided by either ‘creating a sense of belonging' or ‘envisioning prosocial potential'. Through these behaviours, they either navigate within given organisational structures or enact new ones.

By emphasising an agentic view on employees in relation to CSR, we contribute to the literature in two ways. First, prior research on CSR and work meaningfulness has primarily focused on how the mere awareness of organisational-level CSR contributes to employees' experiences of meaningful work, while recently, scholars have called for more research that considers the active role of recipients of CSR activities that enhances the meaningfulness of work (Glavas, 2016; Aguinis and Glavas, 2019; Girschik et al., 2022). Our research underscores the agency that employees have in shaping their experiences of meaningful work, vis-à-vis their experience of the organisation's CSR initiatives (e.g., Pratt and Ashforth, 2003; Wrzesniewski et al., 2003). By doing so, we also respond to the call from micro-CSR scholars for more ‘inductively based conceptualisations of the full scope of behaviours that employees engage in with socially responsible intentions' (Seivwright and Unsworth, 2016, p. 3).

Second, prior theorisations of micro-CSR scholars have suggested that a truly fulfilling sense of meaningfulness (i.e., transcendence) results from the strong embeddedness of CSR in an organisation (i.e., integrated within a firm's strategy, routines, and operations) (Glavas, 2012; Aguinis and Glavas, 2013). By considering the role of agency in meaningful work creation, we find that employees can in fact experience meaningful work, even in organisations that have not yet comprehensively implemented CSR in their core operations.

## Theoretical background^1^

### The microfoundations of CSR

Considering that CSR is a multi-level and multi-disciplinary concept (Rupp and Mallory, 2015; Wickert and de Bakker, 2018), scholars have increasingly recognised the importance of studying the microfoundations of CSR to complement existing organisational or institutional-level work (Gond and Moser, 2021). Micro-CSR can be defined as the way employees (including managers) “within an organisation think about and discuss relationships with stakeholders as well as their roles in relation to the common good, along with their behavioural disposition with respect to the fulfilment and achievement of these roles and relationships” (Basu and Palazzo, 2008, p. 124).

Despite a strong increase in publications in this domain, including recent special issues (Jones et al., 2017, 2019), our knowledge of the multifaceted dynamics of CSR and their relationship with employees remains limited (Hansen et al., 2011; Gond et al., 2017; Gond and Moser, 2021). Not the least because a majority of the research is quantitative (Jones et al., 2019). While quantitative studies are generally useful to provide generalizable findings, they are limited in their ability to provide rich insight into phenomena and how these are experienced by local actors ‘on the ground' (Bansal and Corley, 2012). This is why we employed a qualitative approach to get insight into the daily experiences of our informants and their perceptions of the local context. With this study, we advance employee-focused micro-CSR research, that concerns the influence of CSR on employees, further extending micro-CSR research (e.g., Wickert and de Bakker, 2018; Gond and Moser, 2021; Girschik et al., 2022).

### CSR and meaningful work

Our paper departs from existing insights on employee responses to CSR activities as they pertain to the meaningfulness of their work, a stream of research that falls within the domain of micro-CSR. Broadly speaking, meaningful work reflects individuals' experience of their work being personally significant and worthwhile (Lysova et al., 2019), and it should be differentiated from work meaning that captures what work signifies (e.g., just a job, a calling, etc.) (Rosso et al., 2010). Although there are different ways or mechanisms through which individuals come to see their work as meaningful (for review see Rosso et al., 2010), in this paper, we focus on the process that reflects Pratt and Ashforth's (2003) differentiation between meaningfulness *in* work (i.e. meaningfulness derived from what one does) and meaningfulness *at* work (i.e., meaningfulness derived from membership and identification with a valued group or an organisation). In this paper, due to the focus on a healthcare context, we are concerned with the notion of being able to make an impact with one's work and a feeling of a sense of belonging as particularly relevant to the experiences of meaningful work. Both making an impact and experiencing a sense of belonging are seen as two important pathways or components of meaningful work suggested by Lips-Wiersma and Morris (2009). The notion of impact or contribution to a greater good is important for work to be purposeful and, therefore, meaningful (Martela and Pessi, 2018). It has been also discussed in the job design literature, focusing on the perceived impact of beneficiaries – people or groups of people that are positively affected by one's work – how it fosters experiences of meaningful work (Grant, 2007). Belonging – “a sense of being part of something larger than the self” (Schnell et al., 2013, p. 546) – enables meaningful work because it embraces identification or membership with desirable social groups and provides affective experiences of interpersonal connectedness (Rosso et al., 2010).

Many contextual factors (e.g., job design, leadership, etc.) were found to foster experiences of meaningful work (Rosso et al., 2010; cf. Lysova et al., 2019). Among these factors, recently attention has been paid to the role of CSR. Research argues that CSR triggers positive meaning-making of work and fosters meaningful work (e.g., Pratt et al., 2013; Aguinis and Glavas, 2019; Lysova et al., 2019) because it enables employees to contribute to prosocial goals they care about (Aguinis and Glavas, 2012) and extends their understanding of work beyond the boundaries of their specific job and organisation (Aguinis and Glavas, 2019).

While scholars have emphasised the importance of combining the domains of meaningful work and CSR (e.g., Rosso et al., 2010; Aguinis and Glavas, 2012; Glavas, 2016; Jones, 2019), research has mainly been conceptual (e.g., Michaelson et al., 2014; Aguinis and Glavas, 2019; Lips-Wiersma, 2019; Opoku-Dakwa and Rupp, 2019) and empirical research addressing the two remains limited (Opoku-Dakwa and Rupp, 2019). In their review of the literature, Jones and Rupp (2017) found one quantitative study that considers the effect of external CSR (i.e., CSR directed at stakeholders outside the organisation) on work meaningfulness (i.e., Glavas and Kelley, 2014). They propose that “the opportunity to find personal meaning through an employer's CSR, we believe is a promising avenue for future research” (p. 345). Overall, the few empirical studies that explicitly have focused on examing the relationship between CSR and meaningful work, have mostly adopted a quantitative approach (e.g., Glavas and Kelley, 2014; Raub and Blunschi, 2014; Brieger et al., 2020). The literature would benefit from qualitative studies, since they are better equipped to explore individuals' subjective experiences at work and how they find meaning in the context of CSR (Aguinis and Glavas, 2019).

### Employee proactive behaviours in the domain of CSR and meaningful work

We find that research has focused on how the mere awareness of CSR can be a source of meaningfulness. For example, suggesting that CSR can increase employees' perceptions of being part of a firm that serves a higher purpose (Glavas and Kelley, 2014), can provide a sense that justice is being done to others (Rupp et al., 2006), and allows for a sense of trust (Hansen et al., 2011) and identification with the firm (Kim et al., 2010). Our research is motivated by this prevailing assumption in the literature on CSR and meaningful work that employees are rather passive recipients of their organisation's CSR activities (Aguinis and Glavas, 2019). This is problematic because it underemphasises the agency individuals may have to enact and shape CSR and their work to enhance the meaningfulness of work (Aguinis and Glavas, 2019; Opoku-Dakwa and Rupp, 2019).

We follow the suggestion that the individual pursuit of meaningful work may be best understood as a dynamic and ongoing process, characterised by the importance attributed to subjective meanings and interpretations that lead individuals to discover the significance of their work (Mitra and Buzzanell, 2017; Lysova et al., 2022). Considering that (positive) meaning-making of work is a subset of sensemaking (i.e., the process through which individuals assign meaning to aspects of life, including work) (Weick, 1995), we would expect that employees could actively reflect on CSR in relation to the meaningfulness of their work and that these interpretations would function as cues for further action (Weick, 1995). With this study, we move beyond the perspective of the employee as a passive recipient in the context of CSR, and follow recent suggestions to take an agentic perspective regarding how we view and study employees and their meaningful work experiences in the context of CSR (e.g., Aguinis and Glavas, 2019; Girschik et al., 2022). More specifically, we explore the behaviours through which employees shape their meaningful work experience vis-à-vis their experience of the organisation's CSR initiatives by addressing the following question: How do employees experience and react to CSR initiatives in their pursuit of meaningful work?

It has further been suggested that only under the right circumstances CSR is likely to lead to meaningful work. Aguinis and Glavas (2013) argue that only when CSR is embedded (i.e., “when it involves an organisation's core competencies and integrates CSR within a firm's strategy, routines and operations”, p. 314) – as opposed to peripheral – it is likely to lead to meaningfulness at and in work. We extend these conceptualizations by empirically exploring whether employees can indeed only find work meaningfulness in organisations that embed CSR. Namely, as employees can proactively shape their work to make it more meaningful (Wrzesniewski and Dutton, 2001), they could potentially do this in their job, as well as in their work environment, and experience meaningful work in organisations that do not embed CSR. By taking an agentic perspective, we are able to illuminate the behaviours through which employees overcome perceived misalignments in the implementation of CSR and enhance the meaningfulness of work. Considering that it has been suggested that only when CSR is embedded, employees can find a true sense of meaningful work (e.g., Aguinis and Glavas, 2013), this contribution sheds light on the fact that employees can experience work meaningfulness, even in organisations where CSR is a work in progress. Understanding how employees find meaningful work in these organisations is important because embedded CSR remains something that most organisations aspire rather than fully implement.

### CSR in healthcare

To understand employee reactions to CSR in the healthcare context, we must depart from the general meaning of CSR as an umbrella term (Pedersen, 2010; Wickert and Risi, 2019; Brown et al., 2022) and identify the specific characteristics of CSR in healthcare. Scholars have called for more research that investigates CSR in this context (e.g., Russo, 2016; Crane and Matten, 2021). Russo (2016) argues that understanding CSR in healthcare requires the realisation that “as many practices in healthcare are already socially responsible, progressing from a series of socially responsible behaviours to a socially responsible organisation entails a more consolidated awareness of the health sector's mission and the needs of its participants” (p. 323). Thus, we must distinguish between social behaviours that are required for a functioning healthcare organisation and what activities feed into structurally building and retaining a socially responsible organisation (i.e., CSR).

Following the language of our informants, we apprehend CSR in this context to be (a) focused on the needs of residents (and their families); (b) focused on the needs of employees; (c) implicit in the sense that employees do not use this term; and (d) people-oriented organisational responsibilities that are prioritised over responsibilities that focus on the ecological environment. Meaning that, CSR in healthcare is not limited to, but should first and foremost have, a focus on its social and ethical impact on society and its participants (Russo, 2016). In sum, CSR in healthcare focuses on satisfying the needs of its key participants now and in the future, through ‘shared governance, personal and professional responsibility, a holistic approach in medicine and cooperation for the corporate good as well as for the health of the patient' (Russo, 2016, p. 332). This definition puts a strong emphasis on the social side of the concept, and the environmental side of CSR does in fact remain underemphasized compared to more general definitions of CSR (e.g., Dahlsrud, 2008 and Jamali, 2008).

What is more, although work in healthcare is objectively seen as meaningful, with the latest developments in the industry in terms of regulations and commercialisation (Lake and Friese, 2006), even the meaningfulness of healthcare workers is challenged. Considering that healthcare organisations are dependent on employees being able and willing to continue doing their work (Both-Nwabuwe et al., 2018), it is crucial that we gain a contextual understanding of how individuals construct meaningful work (Lysova et al., 2019).

## Methods

To understand how employees experience and react to CSR initiatives in their pursuit of meaningful work we conducted a comparative study based on two cases (Yin, 1994) because it enables us to compare insights from different organisational contexts (Miles and Huberman, 1994). For the data analysis, we use a grounded theory approach (Glaser and Strauss, 1967; Gioia et al., 2013), since we aim to develop theory by illuminating how individuals interpret and make sense of the daily realities in which they participate (Suddaby, 2006), and how they act on these interpretations. Grounded theory enabled us to elevate the data from descriptive observations to a conceptual level. Overall, our research process was iterative, moving between data and theory. This enabled a relevant theoretical grounding in both the literature and our data.

### Research context

The two case organisations focus on a wide spectrum of elderly healthcare. In the healthcare sector, employees often feel a ‘calling' to do their job, meaning they experience a deep sense of meaningfulness in doing the work as they see it as their duty or destiny (Bunderson and Thompson, 2009). For these individuals, contributing to the ‘greater good' at work is particularly important (Colby et al., 2001) because it allows them to pursue a meaningful life (Bunderson and Thompson, 2009). In line with the CSR definition that we use – focusing on the roles, relationships and behaviours of organisational members as it pertains to stakeholders and the common good (Basu and Palazzo, 2008) – our sample thus serves as an illustrative case in which employee reactions to CSR are particularly salient (Eisenhardt and Graebner, 2007). Below, we describe the two case organisations and the selection process of the cases.

The selection of the two cases followed six explorative interviews with experts. The purpose of these interviews was 2-fold: In the first place, these conversations directed our attention to the importance of this topic in the healthcare sector, and enabled our understanding of the organisational differences that affect employees' perceptions of CSR and meaningful work. This information informed the selection of the two case organisations (see Table 1). Based on these interviews and the literature, we selected cases that differed in their CSR profile and size. For example, in the preliminary interviews, a manager of an association for healthcare organisations observed that CSR was more often part of the organisational strategy in the larger organisations, compared to the smaller organisations. We expected these organisational differences to affect the phenomenon of interest of this study. More specifically, we expected employees to find either meaningfulness at or in work as a result of different CSR profiles in the case organisations (e.g., Pratt and Ashforth, 2003; Aguinis and Glavas, 2013). Overall, the selected cases were similar enough to be compared based on their products and services, and different enough to be compared on the study's contextual factors of interest. Through these interviews we were able to identify potential organisations for our study and gain access to the relevant people in these organisations. Second, these interviews helped us gain an initial understanding of the dynamics of the phenomenon of interest and to develop an interview protocol.

**Table 1:** Summary of major characteristics of case organisations.

**Characteristics**	**Well care**	**Plus care**
Products and services	Elderly homes; all with restaurant also open to non-residents and a small supermarket; some locations have a café, gym or general practitioners practice in-house.	Elderly home; with restaurant, also open to family of residents.
	Nursing-at-home service; in two cities	
Number of employees and locations	Two thousand employees, four locations	200 employees, one location
CSR profile	Focus on social and environmental sustainability; CSR is explicit (mission statement on website); CSR initiatives include changes related to energy use and buildings; installing an employee health and vitality manager	Focus on social sustainability (employees, residents and neighbourhood community); CSR largely implicit (CSR mission statement released only recently, not at the time of interviews)
Organisational structure	Traditional, hierarchical: management team, with location managers and line-managers	Flat: Self-organised teams and a management team
Public ratings and awards	9,2 out of 10 on zorgkaartnederland.nl (residents rating Dutch healthcare organisations), shortlisted for the national award for outstanding healthcare	7,5 out of 10 on zorgkaartnederland.nl (residents rating Dutch healthcare organisations), has increased to 8,5 since new management was installed in 2018 (around the time of interviews)

The first case, Well Care^2^, is a privately held healthcare organisation that offers a wide range of services to elderly people in the Netherlands, including four elderly homes (each with 100–300 residents) and a nursing-at-home service. The organisation offers communal living rooms and restaurants to serve residents and the neighbouring community. With more than 2,000 employees and volunteers, it was the largest organisation in our sample. The organisation has a top management-driven approach to CSR, with CSR being implemented mostly by and at the strategic level. In Well Care, CSR is embedded in policies, leadership commitments, its focus on reputation, and an explicit CSR mission statement, which states the following:

“Well Care focuses on the regional society to make an impact. We want to contribute to healthcare, as well as to the world around us. From a strategic point of view, we want to deliver a measurable contribution to sustainability issues, which we have divided in the 3 P's: People, Planet, and Profit. […]” (Fragment of the CSR mission statement taken from the Well Care website).

Furthermore, Well Care is well-known for outstanding resident care that goes well-beyond the basic requirements for elderly care, both according to the organisation and external sources – Well Care has won a price for this in a national competition. In line with this social image, we reasoned that employees would experience high levels of meaningfulness *at* work, while the lack of opportunities to contribute to CSR might limit possibilities for them to experience meaningfulness *in* work. We interviewed 17 Well Care employees from two locations and different disciplines (see Table 2 for more details), including nursing and non-medical caretaking staff, and two operational managers.

**Table 2:** Data sources and use.

**Data source**	**Number**	**Type of data**	**Use in the analysis**
Interviews (182 pages, Single-spaced)	30	*Preliminary interviews (5)* with experts on HRM and sustainability in the healthcare sector. Two managers of regional associations for healthcare organisations, one board member of healthcare organisation, one manager from NGO, and one career counsellor	To become familiar with issues in the field that are related to the phenomena of interest for this study, and to select appropriate cases.
		*Focused interviews (25)* with medical and non-medical employees who are involved with providing (health)care to residents. Among them, three were operational managers	To investigate processes through which CSR affects meaningful work and identify resulting agentic behaviours.
Observations	9 h	*Field notes from nonparticipant observations in communal living areas (six different days)*. Written record of social interaction among employees, managers, and residents, and activities	To inform interview questions, provide context to the data, and ‘triangulate' the information in interviews.
		*Informal conversations*. Informal talks with managers, and employees engaged with care. Ranging from brief exchanges to longer and very personal conversations, and panel-like discussions in group setting (the latter were recorded, one-to-one talks were not)	To become familiar with organisational context, build trust from informants, address a lack of clarity from observations or interviews, or ‘test' emerging interpretations.
		*Pictures*. Visual documentation of the organisational environment, including written messages from management that appear in work/living areas.	To provide context to narratives in data about communication from management to employees.
Archival data		*Annual Reports:* Of Well Care (2015–2018). Including short- and long-term strategy reports on client-oriented CSR	To become familiar with the organisational context and aid the interpretation of findings.
		*Websites:* Detailed screening of Well Care and Plus Care's websites	To illustrate the preferred external (social) image of the organisations.

The second case, Plus Care, is a relatively small organisation that has ~200 employees and volunteers, and provides services similar to those of Well Care. The organisation takes an employee-driven approach to CSR, in the sense that initiatives emerge through cooperation between employees and management and are largely implemented at the employee level. This is, for example, reflected in the availability of resources for employees to engage with CSR, the perceived effectiveness of the policy on the work floor, and employee involvement in decision-making. In line with this, we expected that the employees would experience high levels of meaningfulness in work (i.e., through a sense of impact).

2 years prior to this study, Plus Care implemented ‘self-organised' teams to enable employee impact on resident well-being. This was the organisation's solution for increasing its social responsibility standards, improving its reputation accordingly, and decreasing employee turnover that the organisation had been struggling with in the past years. This transition to self-organised teams, however, was implemented *ad-hoc*, as also stated by the new management (instated 3 months before the start of this study) in their annual quality report:

“Two years ago, much change was needed in order to be a stable organisation again. […] Much money was invested, for example, in hiring new employees. Eventually, the organisation has become increasingly organised again, with a clear division of responsibility and labour” (Plus Care quality report, 2019).

During the study period, Plus Care did not have an explicit CSR statement. However, towards the end of the year, one was formulated: “At Plus Care we find sustainability of paramount importance. We invest in sustainable employment of our employees but our sustainability also shows in how we handle energy use. […]” (Plus Care Website). At Plus Care, we interviewed seven employees from different disciplines.

These organisational profiles offer a unique opportunity to study the process of finding meaningful work in the context of different approaches towards CSR, being either top management-driven or employee-driven. Furthermore, the two case organisations provide opportunities for employees to find either meaningfulness at or in work (Pratt and Ashforth, 2003).

### Data collection

Our main source of data was semi-structured interviews. We selected a range of employees with a variety of functions and educational backgrounds. When talking about the beneficiaries of their work, our informants preferred to refer to residents as “residents” or “clients”, and avoided the term “patients”. Therefore, we hereinafter use the term resident. We conducted a total of 25 interviews in the two case organisations to gain retrospective and current accounts of participants' experiences regarding the organisation's social profile and their subjective experience of work (Gioia et al., 2013). Next to some fundamental questions about what drives employees in their work (meaningful work), we asked employees how they would describe the organization's mission, how this affected them, and how they contributed to this mission through their own work. We further asked what hindered them to contribute to the organisation's social mission, how this affected their own work experience, and how they dealt with that.

At Well Care we interviewed two line managers, who each managed 3–4 different teams. These line-managers worked in close proximity to the residential area, and they were engaged in the planning of care of residents, next to their managerial and administrative tasks. Plus Care, being smaller and having self-organised teams, did not have line managers. At Plus Care we interviewed the manager who was responsible for all health-care and residential-related topics. This manager knew the individual residents but had a more distant role than the line managers of Well Care. In both organisations, our emphasis was on doing the interviews with employees who worked with the residents daily. Due to regulations of the sector, their roles and job descriptions were similar in both organisations, and we made sure to include participants from a variety of functions in both organisations. These roles included nurses, non-medical caretakers, and activity counsellors. Employees with the function of nurse and non-medical caretaker were responsible for the daily care; medical (e.g., giving medication, treating wounds) and non-medical (e.g., washing, dressing). Based on their education, employees were allowed to engage in certain (medical) activities or not. Activity counsellors worked in the communal living area of the group and organised daily activities for residents (e.g., tea and coffee, cleaning, entertainment). We further interviewed three interns who had been working at the organiation for multiple months. Most participants were female (21), as is representative of this sector (Both-Nwabuwe et al., 2018). In Well Care, participants had worked in the organisation for an average of 13 years, and in Plus Care the average tenure of the participants was 12 years. The interviews lasted 45 min on average and were all recorded and fully transcribed.

For both sample organisations, we followed a similar approach to recruit participants: based on conversations with management and line-managers, the first author was granted access to collect data in four different teams in Well Care, and organisation-wide in Plus Care. Employees were informed about the presence of a researcher and the purpose of the research both through the employee newsletter and in person by their line manager. The first author sat down in the communal living areas of the residents of these specific teams, which was also the central work area of employees when they were not on rounds. This approach allowed employees to get familiar with the researcher in an informal way, and to signal when and if they were open to sitting down for an interview. At the same time, it gave us an extra opportunity to observe the dynamics in the team and their interactions with residents, which also helped us ask relevant probing questions during the interviews.

The data was coded by the first author and each step in the process was checked and discussed regularly with the other authors to deal with possible researcher bias. Next to that, the first author kept a research diary to reflect on her own assumptions as a researcher and she presented the findings in the two organisations – all of which are suggested as tools to enhance the credibility of the study (Hays et al., 2016). Additionally, we used other data sources for this study. We conducted 9 h of non-participant observations, both in communal spaces of the organisation (e.g., central hall) and the (communal) living space of residents when the employees were at work, resulting in 6 h at Well Care and 3 h at Plus Care. While the in-depth interviews were our primary source of data, field notes from observations provided context to informants' narratives and informed our interview protocol. Moreover, we analysed annual reports from 2015 to 2018 and other external communication tools (e.g., websites) to determine how the organisations externally communicate their social values. See Table 2 for an overview of data sources and how they are used in the analysis.

### Data analysis

The nascent state of literature on the CSR–meaningful work relationship calls for an inductive research approach (Suddaby, 2006). We used established procedures of grounded theory (Glaser and Strauss, 1967) to analyse our data and to develop theoretical insights by illuminating the socially and individually constructed processes through which individuals interpret and react to their daily realities.

In a first-order analysis, we engaged in within-case data analysis where we assigned a broad array of codes to the data in words that resembled those of the informants (Gioia et al., 2013). In this phase, we specifically looked for instances where people reflected on the subjective experience of their work and of their organisations' CSR activities (e.g., leadership is committed to CSR goals) as well as employees' reactions to these subjective experiences (e.g., prosocial rule-breaking to enhance positive impact), which present our first-order order concepts. In line with the constant comparison method (Glaser, 1965), we embarked on preliminary analysis during the data collection phase to inform subsequent interviews (Gioia et al., 2013). Well into the first-order coding, having become more familiar with the emerging themes, we used tables and graphs to aid the interpretation process and identify relevant theoretical concepts in each organisation (Miles and Huberman, 1994).

For the second-order analysis, we reduced the number of codes through axial coding – looking for differences and similarities among the codes (Gioia et al., 2013) – and merged them into more general categories. Only well into the second-order analysis, we consulted the literature on micro-CSR and meaningful work to inductively listed interpretive themes (Miles and Huberman, 1994). We further engaged in cross-case analysis to compare processes and themes among the cases (Eisenhardt and Graebner, 2007) and it enabled us to interpret differences in findings among the cases.

At this stage, we noted that employee perceptions of CSR differed between the cases (top-management driven CSR approach, employee-driven CSR approach, and misalignment), and identified different proactive behaviours that employees engaged in to enhance their work meaningfulness (reshaping work for impact, collectively enabling impact, creating a sense of belonging, and envisioning prosocial potential).

At the highest level of analysis, leading to aggregate dimensions, we aimed to work towards higher dimensions. This, for instance, led us to the classification of the behaviour in two abstract dimensions: (1) creating positive impact and (2) creating a sense of meaningful membership.

**Figure 1:**
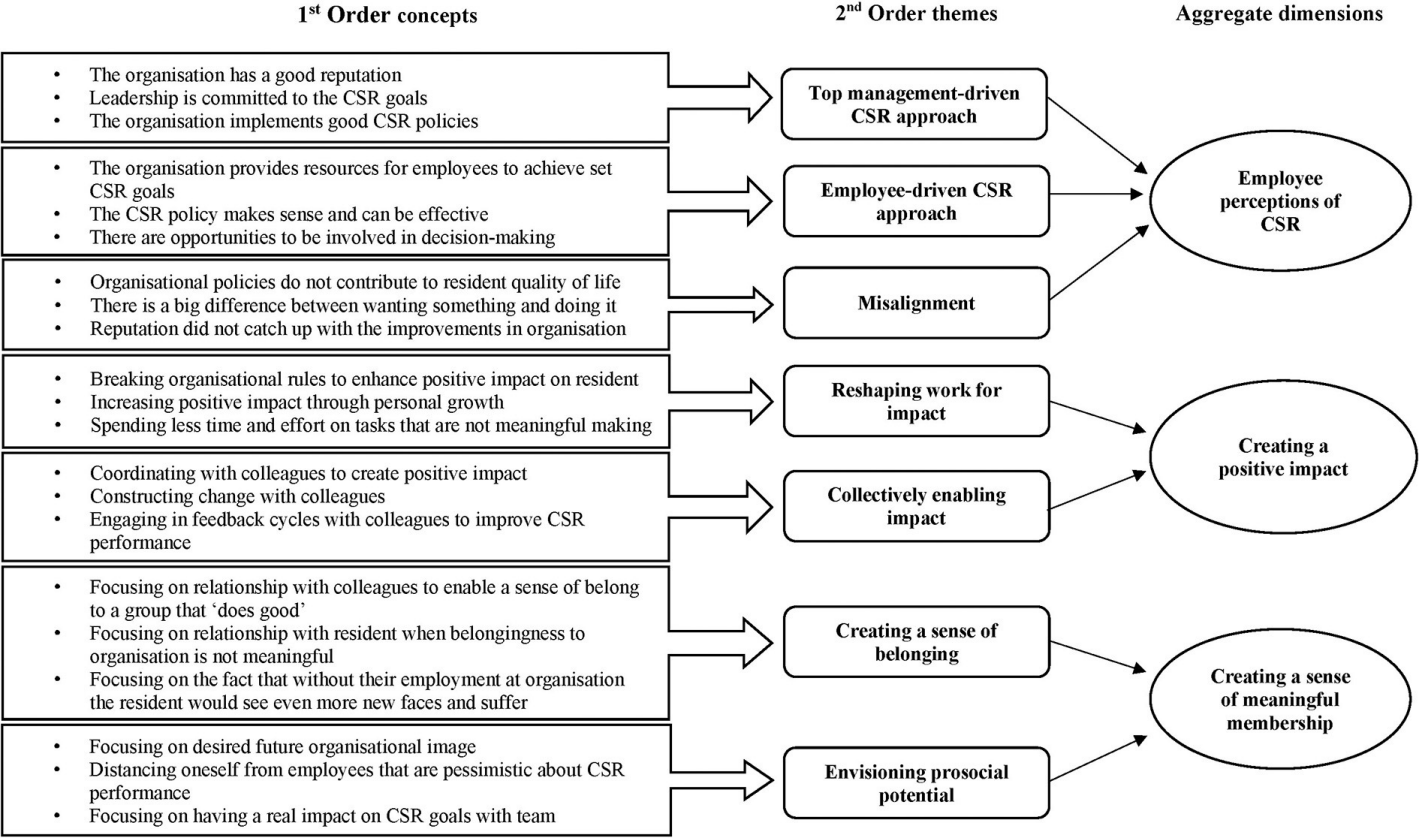
Data structure.

Even though it is not the main source of data in this analysis, we employed archival data such as annual reports and the organisations' website, and also used observations (e.g., at company presentation about vision regarding work and sustainability) to both triangulate and interpret statements of our respondents. Overall, our research process was iterative, moving between data and theory. This enabled a relevant theoretical grounding in both the literature and our data. Figure 1 provides overview of the data structure.

## Findings

We found that the distinct CSR profiles of the case organisations triggered employees to make sense of the meaningfulness of their work, as they incited employees to (re)define what is personally important to them. By CSR profile, we mean the portfolio of strategies, commitments, practices, and procedures of an organisation in relation to CSR, and also, how this organisation presents itself to internal and external stakeholders in relation to CSR. From our data it emerged that most employees of our sample experienced a misalignment between the strategic implementation of CSR and employee-level implementation of CSR, which constrained the meaningfulness of their work. Employees in both cases then proactively shaped their job and work environment to infuse their work with meaning. The significance of the agentic perspective presented here became salient from our data, enabling us to explore how employees could still experience work meaningfulness in organisations where they experienced this CSR misalignment.

Below, we first provide a descriptive account of employees' understanding of CSR in healthcare. We then illustrate how they perceive CSR in their respective organisations, focusing on the misalignment between the strategic implementation of CSR and employee-level implementation of CSR, and outline how this influences employees' meaning-making of work. Lastly, we present a typology of four meaning-infusing behaviours through which individuals try to proactively deal with the experienced misalignment in the implementation of CSR to experience meaningful work.

### Employee perceptions of CSR

Employees in both cases conveyed an implicit understanding of CSR and referred to their organisation's socially responsible policies and practices, as opposed to them using the term CSR. They suggested that the social responsibility of their organisation should focus on two main groups: (potential) residents and their families. For them, being socially responsible as a healthcare organisation meant going beyond the provision of adequate care to further contribute to people's quality of life both inside and outside the organisation. This meant putting residents' well-being first instead of merely providing basic healthcare with a strong focus on profit (e.g., as reflected in an organisational emphasis on efficiency), as described by the employee below:

“Here, they listen to and focus on the resident. And that is important, that the resident is the main focus. […] There is more focus on the resident and what the needs of the residents are. In the past it was only washing, dressing, eating, and you did not see the resident any more than that during the day, and now it's a full 24-hour picture”. (Loes, Well Care)

Furthermore, CSR policies and practices directed at employees played a crucial role in the perceived social responsibility of the organisations. Examples of employee-focused CSR include programmes regarding healthy food, work–life balance and the prevention of burnout; see an excerpt from Well Care's 5-year policy plan below:

“Stimulating an active lifestyle and good nutritional habits have a positive effect on the health of employees. This enables the continuity (of care) and leads to lower absenteeism, and outstanding functioning of employees and teams”. (Well Care, policy plan)

Employees mentioned that these practices were crucial to enable a standard of care that they considered socially responsible, for two main reasons: (a) to secure the continuation of care (i.e., having enough employees willing and able to do the job), and (b) to safeguard the quality of life of residents, who are vulnerable and dependent on the efforts of employees. One employee explained that the core meaning of his work is to safeguard the health and well-being of residents - which captures the meaning of CSR in healthcare – and that employees' suffering at work will inevitably lead to suffering for the resident:

“I find it really important that you are important to an organisation. If that is not the case, you feel you are only a number and you do not feel listened to. You start suffering from that and, indirectly or directly, the patient suffers from that as well. Which is not what you want. That [the well-being of the resident] is what you want to safeguard above all else”. (Eloy, Well Care)

In sum, CSR perceptions of employees were shaped by the organisations' practices and policies directed at (potential) residents inside and outside the organisation, their families, as well as the employees. Employees considered the core meaning and purpose of their work and of the organisation to work towards these social goals.

### Misalignment between strategic and employee-level implementations of CSR

We found that perceptions of CSR can trigger the meaning-making of work in both a positive and negative sense, and this depends on whether employees perceive a misalignment – which we will elaborate on here. Employees considered Well Care to have a top management-driven approach to CSR, with a strong strategic implementation of CSR (e.g., as showcased in its organisational reputation, leadership commitments, and policies), but to lack CSR implementation at the employee level. Plus Care, by contrast, was considered to have an employee-driven approach to CSR (e.g., reflected in the availability of resources for prosocial goals, the effectiveness of the policy on the work floor, and opportunities to be involved in decision-making), but to lack comprehensive CSR implementation at the strategic level. As a result, employees in both organisations perceived a misalignment, as discussed in the following subsections.

#### Well Care

**Figure 2:**
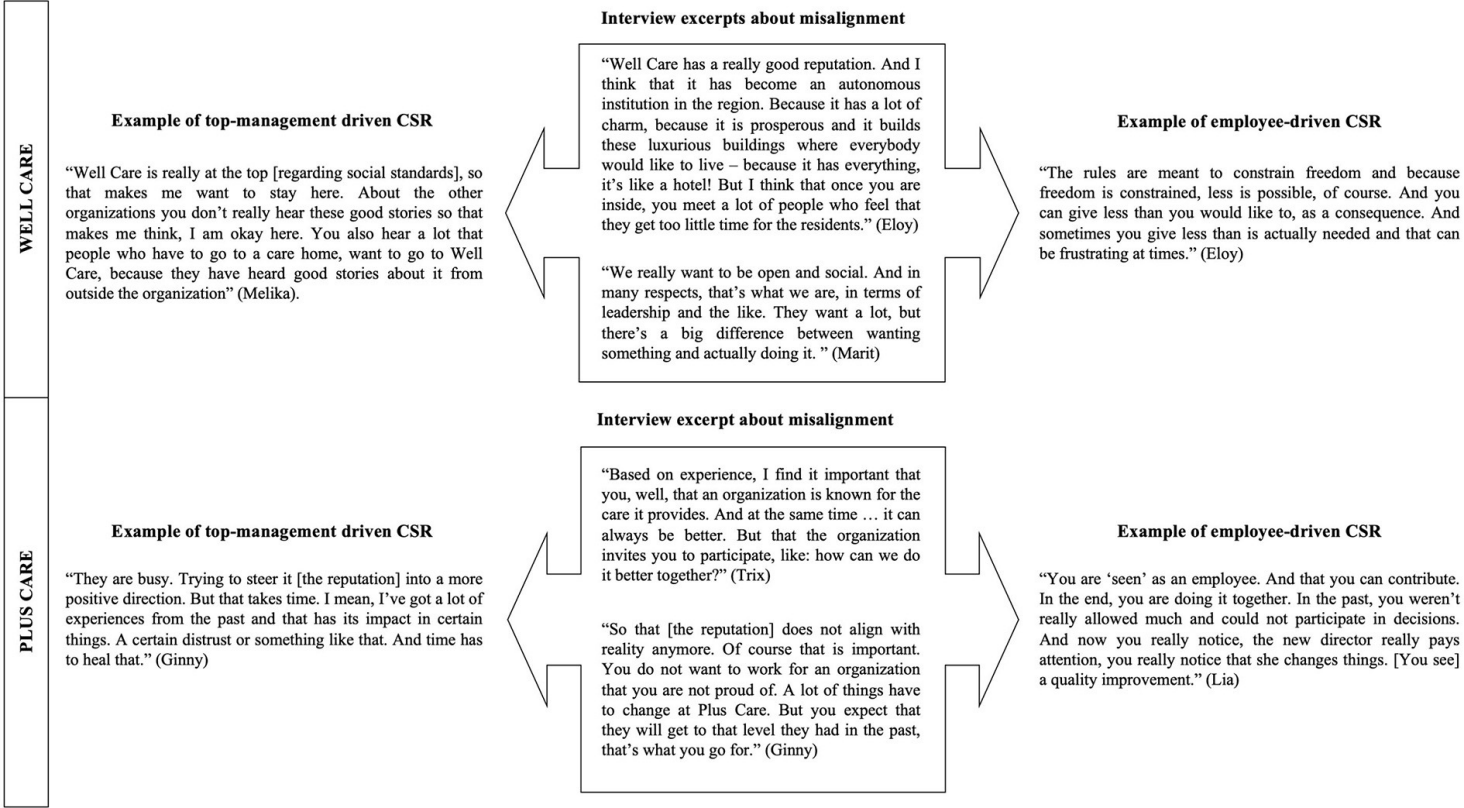
Interview excerpts about misalignment.

While Well Care employees were proud to be part of an organisation with a strong prosocial reputation based on an explicit CSR policy and felt part of an organisation that ‘does good' as a result of their leader's vision (see examples from the data in Figure 2), they missed opportunities for personally making a meaningful contribution through their work. Well Care's approach to implementing CSR top-down is showcased in their written policy plan, in which they state that they will be an ‘outstanding' organisation by going above and beyond in caring for residents and employees. The excerpt below highlights the part that shows how they aim to achieve this, namely, through the top-down implementation of protocols and rules:

“In 2020, protocols and procedures are fully integrated in the daily work. The policy is evaluated yearly by the management team on the basis of yearly plans and management reviews by the departments”. (Policy plan, Well Care)

An employee talked about a prosocial goal of the organisation, stating that “there is a lot of talk about experience-oriented care”. Which refers to looking beyond health and considering the richness and quality of life at all stages of life. He finds, however, that the organisation's policy and rules hinder employees from living up to the organisation's prosocial goals, which triggers him to redefine his sense of work meaningfulness:

“I have the impression that this is not always lived up to in practice. Because, you just notice that there are so many rules, that everything is planned and structured, that there actually remains very little for this experience, for the person itself. […] Sometimes you give less than is actually needed and that can be frustrating”. (Eloy, Well Care)

Employees expressed that the organisation's CSR image should be reflected in their daily work. The quote below shows how an employee felt the organisation is unworthy of a good reputation as long as its employees cannot make meaningful contributions through their work. Not enabling employees contributing to the prosocial goals of the organisation – for example, by including them in decision-making – made employees doubt whether CSR was a genuine social pursuit of the organisation. Moreover, employees saw themselves as important judges of the actual state of social responsibility in the organisation, as they consider themselves vital actors when it comes to implementing it and observing its results on a daily basis:

“When I cannot deliver satisfactory healthcare. I am of the opinion that such an organisation does not deserve to have a good reputation. Because eventually I find that, as an employee, you see what the quality of healthcare actually is, because we are the people who actually provide the care. So, I think that employees are more of an indicator of good healthcare standards than a manager or someone else, someone from the management team or something”. (Lisa, Well Care)

In sum, Well Care has a top management-driven approach to CSR. As a result of the misalignment that employees experience between the strategic implementation of CSR and the personal contribution they can make through their work, they struggle to experience work meaningfulness. Figure 2 provides examples of employee interpretations of CSR and the perceived misalignment.

#### Plus Care

At the time of the interviews, Plus Care had recently changed leadership. A CSR vision had not yet been formulated or communicated and, according to the employees, the organisation's social reputation had not yet caught up with the new reality. Employees struggled to make positive meaning out of their work as a result of a troubled reputation and lack of a socially-oriented vision. However, they found that the new leadership provided employees with opportunities for contributing to CSR within the organisation. They perceived CSR as being implemented at the employee-level, for example, because of the availability of resources to contribute through their work (e.g., having enough time to spend with residents) and being included in decision-making that would impact their ability to reassure high-quality care for residents (e.g., being present at job interviews for new employees; see examples from the data in Figure 2). An employee explained that having these opportunities to make a meaningful contribution as employees, benefitted the well-being of residents:

“It is simply great to organise everything ourselves, with the team. To not constantly have to ask someone “Can we do this, are we allowed to do that?” This autonomy, I think it is stimulating. […] And you see this reflected in the well-being of the residents”. (Ginny, Plus Care)

Although employees were able to make a meaningful social contribution through their work, explicit CSR measures were lacking in the organisation. Employees explained that this lack of organisational vision for CSR affected the reputation of the organisation. The employee below explains how the decline of the organisation's social reputation, specifically, regarding the ability to provide care that she considered socially responsible, affected her sense of self-worth and organisational pride – important elements of work meaningfulness:

“It is nice to work for an organisation with a good reputation. Plus Care's reputation has declined. Before, it was the residence, you had to go there. People also talked very positively about it in the region. […] That has changed enormously. They are saying that “At Plus Care, things are happening that are not appropriate”. So, I noticed that, and that Plus Care no longer has that good reputation. Well, that hurts and I take it personally. You don't want them to talk bad about you, about Plus Care”. (Trix, Plus Care)

In sum, we found that in both organisations, the employees' perceptions of CSR triggered meaning-making regarding the meaningfulness of their work and the organisation's approach to CSR. Specifically, at Well Care, employees felt the top management-driven approach to CSR did not allow them to make a meaningful contribution to CSR. Thus, employee-level CSR implementation was lacking. At Plus Care, by contrast, the employee-driven approach to CSR allowed them to make a meaningful social contribution; however, a strategic implementation of CSR was lacking. This resulted, for example, in the perception that the organisation's CSR image was not aligned with the prosocial impact employees were having through their work. Our data analysis revealed that employees wanted to overcome this discrepancy, and shaped their work in such a way that they experienced some sense of work meaningfulness, despite their perceptions of CSR in their organisations. Specifically, we identified several meaning-infusing behaviours employees engaged in, which are described next.

### Meaning-infusing behaviours to overcome the CSR implementation misalignment

Based on cues extracted from the CSR-related work environment that prompted meaning-making of work, we next describe how this incited employees' subsequent behaviours. We found that employees of both organisations engaged in several proactive behaviours to overcome the aforementioned misalignment and infuse their work with meaning by (a) *creating positive impact* or (b) *creating a sense of meaningful membership*. First, they created positive impact to overcome the misalignment of CSR by shaping the boundaries of the job and work environment. Second, employees pursued a sense of meaningful membership, and reshaped their job and work environment by focusing on particular people or aspects of work that contributed to this. Furthermore, we found that when employees engaged in these behaviours, they either ‘navigated' within given organisational structures or ‘enacted' new ones. Considering the two case organisations, we observed that Well Care employees mostly navigated existing structures, while Plus Care employees more often opted for structural change through enacted behaviours. Additional to the examples presented in this section, more supporting data can be found in Table 3.

**Table 3:** Additional data table for cross-case comparisons: meaning infusing behaviours in the context of CSR.

**Creating positive impact**		**Creating a sense of meaningful membership**	
**Reshaping work for impact**	**Collectively creating impact**	**Creating a sense of belonging**	**Envisioning prosocial potential**
*Well Care – Prosocial rule breaking* “On paper this [protocol] can look great, it might work on an ideal day but in practice it doesn't work. In the end, it's the resident who truly matters and those [the organisation's] rules have to make place for that”. (Melika)	*Plus Care – Engaging in feedback cycles with colleagues* “I have a colleague who was used to medical care, who has been assigned shifts to do day-activities with residents. She struggles with that […] And then they [the colleagues] get involved. Then we talk, listen to each other. Not to immediately point fingers, as in how it should be done, but just to talk about it. And I find that very important. To keep talking. I always say: you can always come to me, if you have problems. We agreed that we would get involved with each other”. (Trix)	*Well Care – Focus on relationships with residents* “I had this feeling at some point, that I did not really want to work here any longer. But then you think, that is sad for those residents. Because there will be even more temporary contract workers and it will be even more chaos”. (Jelka)	*Plus Care – Focus on positive change* “It is important. You are not going to work for an organisation that you are not proud of. [At] Plus Care, a lot has to happen still. But you count on the organisation to get back to the standard and reputation that they had before, that is what I go for. We will get there again”. (Ginny)
*Well Care – Action taking directed at personal growth*. “What brings me happiness at work is that I see above and beyond, and that I strive to develop myself [as professional] and want to keep up with the latest knowledge”. (Loes)	*Plus Care – Assure quality resident care with colleagues*. “Of course, you notice that you have less time for the residents. But together you make sure that they get the care they need. You set priorities. In those periods, that work pressure is high, with shortages of staff, communication with colleagues is crucial. We help each other. You really have to do it together”. (Kelly)	*Well Care – Focus on shared mission with colleagues*. “It mostly is that I see how colleagues [not the organisation] care for residents. Everyone is focused on providing good care. […] To see how colleagues take care of the residents, I find that very inspiring. […] I think that everyone here has the same purpose. They all have that same vision, taking care of the resident in the best way possible”. (Jane)	*Plus Care – Distancing oneself from negative colleagues*. “At the time, a lot of colleagues left. […] I am very down-to-earth about that: I still enjoy going to work and you have to give the change a chance. I can see that, with the changes, [the situation] is improving. So I also find that you have to stand for that. That you have to do it together”. (Didi)

#### Creating a positive impact

To create a positive impact in their efforts to deal with the misalignment in the CSR implementation, employees engage in two meaning-infusing behaviours: *reshaping work for impact* and *collectively enabling impact*. We discuss them below.

#### Reshaping work for impact

Those who engaged in reshaping work for impact to infuse their work with meaning, altered the boundaries of their job and work environment to have more control and impact on residents' well-being, and did so within the existing boundaries of the organisation's established structures. As employees navigated, rather than challenged, existing organisational structures, they shaped the job and work environment without changing their status quo. As a result, this behaviour was not aimed at creating structurally changing what constrained the work meaningfulness in the first place, but did enable employees to have a positive impact on the beneficiaries of their work, the residents. Below, we discuss two examples of this behaviour that emerged from our data.

One illustration comes from the tendency to ignore Well Care's rules and regulations, such as the so-called ‘double medication check' - which refers to two employees having to sign off before administering certain medication to residents. This is a procedure that has been put in place by the organisation to assure resident safety and well-being – an element of their CSR approach. Sometimes the reason for ignoring this, and other rules would be that an employee felt that adhering to them would take too much time. Time that, they felt, could be put to better use in parts of their job they considered to have more prosocial impact. In addition, some rules were even considered counter-effective; that is, such rules decreased the ability of the employee to provide meaningful care for the resident. By reshaping their work tasks and adhering to these rules in a “creative” way, employees enhanced their work meaningfulness without structurally changing the organisational structures that constrained their ability to contribute to CSR and the meaningfulness of work. One employee explained that rules constrained his ability to create meaningful impact, in line with the CSR goal of the organisation to provide experience-based care. When asked how he overcame this barrier, he answered:

“Ignoring rules is not allowed. They want to guarantee the safety of the resident. But, well, it is definitely not efficient, because it is at the expense of the time you could spend on other things [related to the resident], time that you desperately need at other moments. So, what you see is that, at times, employees don't take it too seriously. And then actually, yes, they try to do it in a ‘creative' way. Everyone in their own way. But knowing that there are possible consequences to it”. (Eloy, Well Care)

Another example of reshaping work for impact is the pursuit of personal growth. Well Care offered a wide range of courses for job-related learning and personal development. Employees explained that investing (personal) time in these courses enhanced their potential to deliver outstanding care to residents. When Well Care did not provide the necessary resources (e.g., time) for them to achieve the social responsibility standards they cared about, personal development made them more knowledgeable and efficient, and it was a way to offset the lack of resources provided by the organisation. Ultimately, this enhanced one's potential to have a positive impact on the well-being of residents within the existing structure of the organisation. Employees explained that through learning, they were able to offset doubts such as ‘What am I doing here?' (a question that reflects doubts about the meaningfulness of work), and learning gave them the feeling they could be of greater value to residents. An increased sense of control and impact then resulted in enhanced meaningfulness of work.

“Well Care offers me plenty of study opportunities to grow. If you learn more or study next to your job, then your job gets easier, you get insights into what you are doing, and you learn how to interact with people, residents. These are important points. Otherwise, you feel like, ‘What am I doing here?' I really grew personally”. (Andre, Well Care)

Thus, employees at Well Care reshaped their jobs' boundaries to create opportunities for a prosocial impact on residents, within the existing organisational or team-level structures.

#### Collectively enabling impact

We use the term collectively enabling impact here to capture employees' changing the boundaries of their work environment and their job to make their work more meaningful by increasing their prosocial impact on residents. However, unlike the previously-discussed meaning-infusing behaviour, this kind of behaviour was aimed at changing those structures together with the team. To better illustrate this behaviour, we elaborate on two examples.

First, employees of Plus Care explained how they turn to colleagues to promote CSR goals. At this level, they can contribute to decision-making and positively impact these goals. In the following quote, one employee explains how she and her colleagues took proactive action to increase the positive impact on residents' quality of life beyond their basic healthcare tasks. At the team level they could successfully contribute by developing and implementing ideas, and thereby increasing their sense of meaningfulness in their work:

“Because of the team, I have more time for the residents. I can devote more real attention to them. Colleagues also take over tasks from you. 2 weeks ago, the weather was really nice. We arranged to go on an outing outside together. […] I don't have that feeling anymore of ‘Puh', I worked an entire day. Before, when you worked an entire day, you were tired, done. That is different now”. (Evie, Plus Care)

Another example of collectively enabling impact is the focus on feedback of colleagues to enable the CSR standards together as a team. When asked how she made a positive impact on residents, she explained that (since the new leadership) she turns to the team and that their feedback helps her having this positive impact:

“I just try to make things possible [for residents], no matter what. And if there is something that I cannot do, then I'll ask my colleague or I hand it over to my colleague. And yes, I think they [colleagues] are open for that. If there is something that I'm struggling with, I can raise that, like ‘could we change that?'. They are open for that at the moment. Currently, they are”. (Ginny, Plus Care)

In sum, Plus Care employees created opportunities for a positive impact on CSR goals together with the team, and thereby, enacted existing structures.

Next to changing the boundaries of their work environment and job, employees opted to focus on the aspects of their work environment and job they perceived as meaningful. They altered the way they thought about their job and work environment and focused on elements of the work environment that strengthened their sense of being a part of something ‘bigger than oneself' and an organisation that ‘does good'.

#### Creating a sense of meaningful membership

To create a sense of meaningful membership in their efforts to deal with the misalignment in the CSR implementation employees engage in additional two meaning-infusing behaviours: *creating a sense of belonging* and *envisioning prosocial potential*. In the former, employees navigate the current organisational structures when changing how they think about their work environment and job, whereas in the latter, employees do this in a way that challenged the status quo.

#### Creating a sense of belonging

We use the term creating a sense of belonging to capture employees focusing on specific social relationships that give them the feeling that they did not stand alone in their work and prosocial pursuits. Devoid of a sense of belonging and support of the organisation, Well Care employees shifted their focus away from the organisational level, and instead emphasised the importance of colleagues and residents. From the data, it emerged that they allowed work-home boundaries to blur, in order to have meaningful connexions at work, and experience that they belong, if not in the context of the organisation, then in the context of colleagues and residents. With this behaviour they did not enact the organisational structures that constrained the meaningfulness of their work but instead applied ‘workarounds' to overcome those constraints.

For instance, we found that the need to belong and experience social support in their prosocial pursuit, pulled employees towards their colleagues - which they perceived to be likeminded. Employees also redefined boundaries of family and work relationships, or rather ‘blurred' existing boundaries. This was reflected in the rhetoric that together you are one, and that colleagues are like family. In the following quote, an employee, who explained that membership to the organisation was no longer a source of work meaningfulness, chose to focus on her colleagues instead, both for her own well-being and that of the resident:

“Colleagues give me the feeling that I am being heard and that somebody is looking out for me. Here, at work, we need each other. We are like a small family”. (Iris, Well Care)

Another example of work–home boundaries fading in the search for a sense of belonging can be found in the employee–resident relationship. Considering that employees from our sample might take care of the same residents for several years, it is not surprising that residents can serve as a source of meaningfulness through a sense of belonging. In the void of the organisation providing opportunities to ‘do good' together, within the boundaries of the organisational structure, employees engaged in thinking about and interacting with their residents beyond their job descriptions:

“All employees work overtime hours. As a result, you become even more invested in your resident. Then, you start crossing certain boundaries. Even at the weekend, colleagues are sending each other WhatsApp messages [about residents]. […] they are too invested in their residents, only thinking about them and not looking at themselves”. (Sam, Well Care)

In sum, Well Care employees focused on colleagues and residents instead of the organisation to increase the meaningfulness of their work through a sense of belonging.

#### Envisioning prosocial potential

This behaviour refers to altering the way one thinks about the organisation's CSR and changing the particular social interactions one engages in to increase one's work meaningfulness. By emphasising the potential of a positive future, it is focused on how the current organisational structures and activities can change rather than to accept them. In contrast to the behaviour of Well Care employees as discussed above, Plus Care employees did not direct their focus away from the organisation when their perception of CSR (i.e., misalignment) challenged the meaningfulness of their work. Rather, they sought ways to focus on a positive envisioned future. Below we discuss two examples of this behaviour, namely, employees thinking about the potential rather than current CSR performance, and proactively pursuing social interactions with like-minded colleagues.

Firstly, some framed the organisation's CSR performance as growing rather than currently unsatisfactory. Thus, Plus Care employees altered the way they thought about their *employer* in an equal manner. As a result, organisational membership was perceived as more meaningful. The employee below talked about seeing the potential of the organisation moving towards the prosocial reputation they had in the past, and in explaining why membership is meaningful to her at the moment, she talks about an envisioned future:

“She [new manager] pays attention to the staff, communicates well, and just organises so much. You can just see it, she picks up on things, she is equal to us – that's how I see it. We get updates on the latest news. Lots of meetings. So, you see an upwards move now [in the organisation], in a way that makes you think ‘this is good', and we go for it together!”. (Kelly, Plus Care)

Moreover, employees changed their social interactions and engaged more with colleagues who shared this rhetoric, who were positive about Plus Care's future, and who were willing to work on this together. Accordingly, employees felt contempt towards (former) colleagues who did not support this progress, and actively chose to focus less on them as they were harming the sense of unity they were searching for. For a majority of the employees, a sense of unity and belonging resulted from feeling they ‘were in this together, finding a way out'.

“I see that we have all these new colleagues, good ones. There are a few who left, of whom I thought, I do not mind you leaving. They did not fit in the organisation: trouble makers, a negative attitude, nothing is ever good enough. Then I think, you should leave. […] I think, guys! We are together working on getting back to the historic high standards, catching up, there are new people being hired, so we should give it a chance!”. (Kelly, Plus Care)

Overall, we observed that employees could be successful in their efforts to experience meaningful work even in situations where it is challenged due to a perceived misalignment in CSR implementation. Employees that pursue meaningful work react to their negative perceptions of organisational CSR with behaviours that enable a meaningful work experience when it is challenged. In the following section, we consolidate our findings into a typology of meaning-infusing behaviours.

### Towards the typology of meaning-infusing behaviours in the context of CSR in healthcare

**Figure 3:**
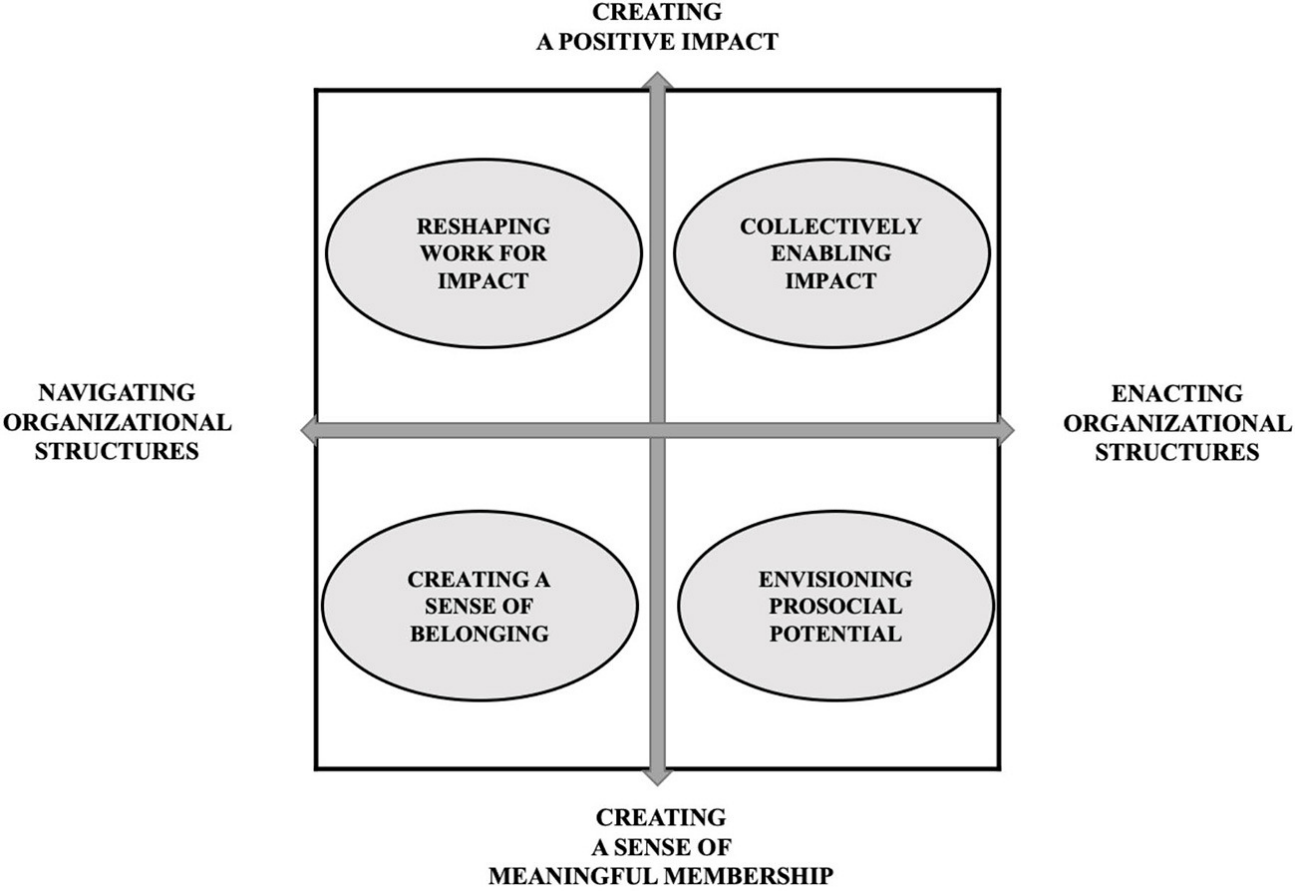
A typology of meaning-infusing behaviours in the context of CSR in healthcare.

Our data revealed that employees engage in different meaning-infusing behaviours and we consolidated these findings into a typology of four meaning-infusing behaviours of employees in the context of CSR, as presented in Figure 3.

The meaning-infusing behaviours of employees can be ordered along two dimensions: (a) whether they aim to create either a positive impact or a sense of meaningful membership, and (b) how they deal with organisational structures. First, on the vertical axis, behaviours are directed at creating a sense of positive impact (i.e., reshaping work for impact and collectively enabling impact*)* or creating a sense of meaningful membership (i.e., creating a sense of belonging and envisioning prosocial potential). This resonates with the Pratt and Ashforth's (2003) differention between meaningfulness in work and meaningfulness at work. Namely, from this, we could argue that the meaning-infusing behaviours of *reshaping work for impact* and *collectively enabling impact* contribute to the meaningfulness *in* work. While *creating a sense of belonging* contribute to meaningfulness *at* work. Next to this, employees engaged in *envisioning prosocial potential*. While it resonates with meaningfulness *at* work, it is more future-focused (Gephart et al., 2010).

Furthermore, Aguinis and Glavas (2013) argued that only if CSR is embedded does it enable experiences of meaningfulness in and at work, which are both required to experience ‘true' meaningfulness, a state also referred to as transcendence (Pratt and Ashforth, 2003). Indeed, our findings are in line with this suggestion. The employees in our sample pursued meaningful work by having a positive impact through their daily work, as well as through a sense of membership to an organisation that strategically implements CSR (e.g., external communication of CSR efforts in the organisation). With our framework we add to the understanding of finding meaningfulness in and at work in the context of CSR, and propose that even when CSR is not embedded, employees who seek meaningful work can still experience it. Our framework provides a nuanced perspective and suggests that when CSR is not embedded, employees engage in proactive behaviours that infuse their work with the significance that they are lacking through making a positive impact or through having a sense of meaningful membership.

Second, as shown by the horizontal axis, we found that the behaviours are either oriented at enacting team- and organisational-level structures or at navigating existing organisational structures. Thus, we show the difference between enacted and navigated ways of altering one's work environment and job. On one side of the horizontal axis, we positioned *enacting organisational structures*. We use the term ‘enacting' to refer to behaviour aimed at changing the way things are currently organised within the organisation. Moreover, our data suggests that employees who engage in this behaviour are more change-oriented than employees who tend to only navigate around existing organisational structures (e.g., such as protocols).

At the other end of the axis, we positioned *navigating organisational structures*. This refers to employees seeking meaningfulness in their work by changing their job within the boundaries of current organisational-level elements that might exert strain on the meaningfulness of work in the first place. Thus, employees accept the status quo and shape their job and work environment by engaging in behaviours oriented towards enhancing work meaningfulness within current structures.

In our framework, we elaborated on the meaning-infusing behaviours that employees engage in to find work meaningfulness in the context of a perceived misalignment in the implementation of CSR at employee- and the strategic level. This framework aims to offer a more nuanced picture of the CSR–meaningful work relationship. Rather than solely considering how organisations can provide opportunities for meaningful work, this framework emphasises the importance of employees engaging in agentic behaviours to contribute to experiencing their work as meaningful in the context of CSR.

## Discussion

In this study, we addressed the question of how employees experience and react to CSR initiatives in their pursuit of meaningful work. Our findings suggest that employees contribute to their own meaningful work experience in the context of CSR by engaging in meaning-infusing behaviours. Our typology of meaning-infusing behaviours thus contributes to the micro-CSR literature concerned with employee evaluations of and reactions to CSR (Gond et al., 2017; Jones et al., 2017). In the following sections, we elaborate on the implications of our research for micro-CSR theory and practice.

### Micro-CSR theory and meaningful work

It has been proposed that CSR triggers employees to engage in making sense of the meaningfulness of their work because it broadens the perception of work to outside of one's particular job and organisation (Aguinis and Glavas, 2019). When we examined extant studies that approach the CSR–meaningful work relationship, we found they typically consider employees as passive recipients of opportunities for meaningful work, while a more active approach is advocated for (Aguinis and Glavas, 2019; Opoku-Dakwa and Rupp, 2019). While prior studies linking CSR and meaningful work have mentioned the importance of agency to the experience of meaningful work (e.g., Aguinis and Glavas, 2019), they have not discussed exactly how or through which agentic behaviours employees might contribute to work meaningfulness in the context of CSR. With this study, we move beyond the employee as a passive recipient of meaningful work in the context of CSR, addressing calls from recent research (Aguinis and Glavas, 2019; Girschik et al., 2022) and instead illuminate how organisations and employees interact in the emergence of meaningful work experiences as a result of CSR. We demonstrate how employees enact their job and work environment when CSR constraints experiences of meaningfulness of work; specifically when employees perceive a misalignment between CSR as implemented at the strategic level and the employee level.

Additionally, this study answers the call to study the behavioural reactions of employees to CSR (Gond et al., 2017). As Gond et al. (2017) showed, most studies have focused on established – and quite general – organisational behaviour outcomes, particularly those of interest to organisations such as organisational commitment (Saks, 2006) and employee engagement (Glavas and Piderit, 2009). Other CSR research has pointed at the specific CSR-related behaviours that employees engage in (e.g., pro-environmental behaviours) (for a review see Girschik et al., 2022). The purpose of this study is not to identify the specific practices through which employees engage with CSR, but rather how they react to CSR more broadly in their pursuit of meaningful work. By taking an agentic perspective, we allow employees to take the agentic role in how we make sense of their behaviours: so not passively reactive but proactively responding to CSR. What is more, while studies on pro-environmental behaviour and CSR engagement often show that these agentic behaviours are typically conducted in line with, and support an already existing CSR strategy (see for review Girschik et al., 2022), we explore behaviours that are not driven by the CSR strategy but by employees.

We uncover specific behavioural outcomes that are triggered by CSR and illuminate how employees enact their job and work environment through agentic behaviours aimed at infusing work with meaning.

### The role of agentic behaviours

According to the meaningful work literature, a sense of meaningful work emerges when both meaningfulness at and in work are experienced (Pratt and Ashforth, 2003; Lips-Wiersma and Morris, 2009). This suggestion has been adopted by micro-CSR scholars studying meaningful work creation (e.g., Seivwright and Unsworth, 2016; Aguinis and Glavas, 2019). We depart from the work of these scholars and expect CSR implementations to affect employees' experiences of meaningfulness in and at work. For example, we expect employees that work for an organisation with a good reputation for CSR to experience meaningfulness at work, due to a felt sense of belongingness or pride (Pratt and Ashforth, 2003). Scholars have further suggested, that only in organisations that apply embedded CSR can employees experience both meaningfulness at and in work. However, we found that employees can still experience both meaningfulness in and at work even when their organisations do not yet employ embedded CSR employees.

Returning to the literature, we realised that in the micro-CSR literature, both meaningfulness at work and in work is considered to emerge mainly under embedded CSR, as there CSR is likely to be implemented at both the strategic level and the daily routines of employees (Aguinis and Glavas, 2013). Instead, scholarship on meaningful work does not require such conditions as it can be experienced even in organisational contexts that are far from perfect (e.g., Bunderson and Thompson, 2009). Our study contributes to the micro-CSR literature through its consideration of how employees find meaningful work in organisations that have not (yet) embedded CSR in all aspects of work and the organisation. This is relevant for both theory and practice, considering that embedded CSR is only realised in a fraction of organisations engaged in CSR, and that a misalignment (as observed in our data) is likely found in the majority of organisations that are in the process of implementing CSR (Aguinis and Glavas, 2013).

The meaningful work literature argues that it is up to an individual to experience meaningfulness in their work (e.g., Lips-Wiersma and Morris, 2009; Lysova et al., 2019). Our data suggest that, in the context of CSR, employees engage in proactive behaviours aimed at shaping or changing their work environment or job to experience a sense of work meaningfulness in the context of CSR. We find that there are behaviours that are aimed at (a)creating a sense of positive impact through reshaping the tasks and boundaries of the job to have more impact on the social responsibility within the organisation more broadly, and the beneficiaries of their work specifically, and at (b) creating a sense of meaningful membership through shaping and adjusting the boundaries of social interactions, or by envisioning the prosocial potential of the organisation. Therefore, individuals showed to take a proactive stance at altering their work environment and job for addressing the misalignment in their CSR perceptions.

Additionally, we distinguish between efforts to shape one's work environment and job by navigating organisational structures or by enacting and changing those structures. We found that employees at Well Care tended to infuse their work with meaning by navigating existing organisational structures such as (CSR-related) protocols, whereas Plus Care employees aimed to create structural change. We suggest that this is due to the size of the respective organisations as well as their different approaches to CSR, namely top management-driven or employee-driven. At Well Care, a large and hierarchical organisation, CSR implementations were top management-driven; employees thus missed opportunities to contribute to decision-making, and some even felt it was not a safe space to speak up. This was reflected in them stating that, for example, having seen colleagues speak up and then leave the organisation, Well Care is a good employer until one disagrees with them. In line with findings from prior research (Morsing and Spence, 2019), Plus Care, the smaller organisation in our sample, actively encouraged employees to speak up and contribute to their CSR. From our data, it emerged that in this context, employees engaged in more structural ways to infuse their work with meaning. Future research can elaborate on this tentative finding by studying how different organisational sizes and approaches to CSR influence the meaning-infusing efforts of employees, testing them in different contexts and further exploring these organisational factors. While we show that due to their meaning-infusing behaviours employees can experience meaningful work in the context of a perceived misalignment in the CSR implementation, we also question how sustainable is this solution for overcoming the misalignment. Future research could explore this matter and particularly for how long and under which conditions employees' proactive meaning-infusing behaviours can enable them to experience meaningfulness in organisations when CSR is not embedded.

### CSR in the context of healthcare

The importance of understanding CSR in its specific domain is recognised only recently (Wickert and Risi, 2019), and our understanding of what pertains to CSR in healthcare is yet to take form (Crane and Matten, 2021). With our inductive design we illuminate employees' perspective on what it means to be a socially responsible healthcare organisation, and aim to contribute to the conversation on what CSR means in different contexts – the contextualisation of CSR – and from the perspective of practitioners in different sectors (Pedersen, 2010; Wickert and Risi, 2019), including healthcare (Russo, 2016; Crane and Matten, 2021). Following the language of our respondents, we propose that CSR in the context of healthcare can be understood as (a) focused on the needs of residents (and their families), (b) focused on the needs of employees, (c) being implicit in the sense that employees do not use this term, and (d) prioritising people-oriented organisational responsibilities over responsibilities that focus on the ecological environment. These findings align with prior suggestions: Russo (2016) outlined how CSR in healthcare should be first and foremost focused on its social and ethical impact on society and its participants (Russo, 2016). Our paper builds on the efforts of Russo (2016) and provides empirical grounding to his suggestions. Moreover, we are driven by our data, and our respondents made little reference to environmental oriented CSR, including pro-environmental behaviours (as opposed to prosocial behaviours). However, this does not mean that it is irrelevant or that employees do not engage in pro-environmental behaviours, rather that it is not salient in our sample. Future research could explicitly study the role of environmentally focused CSR and employees' pro-environmental behaviour in the context of healthcare. Pedersen (2010) further found that managers in different sectors considered their responsibility to employees as an important element of CSR – including the responsibility for dignified work and an inspiring workplace. More specifically, Pedersen (2010) studied managers in healthcare and suggests that “the health-care firm seems to have a very strong focus on product innovation and customer/end-user care” (p. 161). This aligns with our analysis, as we found that employees were focused on the well-being of the resident when they talked about CSR.

### Limitations and implications for future research

This study carries limitations common to this type of qualitative research, which nevertheless open up potential avenues for future research. For example, future research could use longitudinal research methods to provide additional insights into the process through which employees sustain a meaningful work experience over time in the context of CSR (Langley et al., 2013). This could be done, for example, by illuminating what circumstances cause alterations to initial perceptions of a particular CSR activity or policy over time.

Furthermore, we opted for an explorative design and carefully selected and compared two organisations to be able to study in-depth, the phenomena of interest of this study (Gioia et al., 2013). While it is not the purpose of qualitative research to generalise findings, we are aware of certain boundary conditions of this study. Our study was conducted in two organisations in the healthcare sector, one relatively large and one smaller organisation. Prior research has found that indeed the size of the organisation influences employees' perception of CSR (Morsing and Spence, 2019). While we included organisations from different sizes in this study, the focus of this research has been on the individual level – studying how employees engage in proactive behaviours to infuse their work with meaning. The organisational level factors, such as size and CSR profile, were merely the context in which we conducted the study. Future research could focus on the effect of different organisational factors, such as size, and could use qualitative methods to explore the mechanisms through which this influences employees' meaning-infusing behaviours in the context of CSR, or quantitatively test our suggestion about the role of size, autonomy, and perceived CSR implementation on the meaning-infusing behaviours of employees.

Lastly, comparative replications of this study in other sectors could produce interesting results regarding when employees engage in certain meaning-infusing behaviours. Specifically, a similar study could be replicated in a more commercially oriented (or less publicly oriented) organisation that engages in CSR (e.g., retail), as these organisations might also attract employees with greater-good motivations (Jones et al., 2014). Still, we expect that our typology presented in this paper, with its four distinct behaviours, will apply to other organisational settings. Such studies, however, could render insights into the organisational-level influences on specific behaviours employees engage in and consequently illuminate how organisations can facilitate employees' proactive engagement in their own meaningful work creation.

### Implications for management practice

Our findings offer three main insights for management practice. First, organisations recognise the importance of meaningful work for their employees (e.g., Dhingra et al., 2021), not at the least because meaningful work was found to aid the retention and commitment of employees in the organisation, an outcome that is pivotal in today's labour market (Allan et al., 2019). Still, organisations struggle to provide meaningful work to employees, for example, because of the highly subjective nature of meaningful work; what makes work meaningful is very personal, and different for every employee (Lips-Wiersma and Morris, 2009). We move beyond the idea that organisations should be able to readily provide work that is meaningful through their CSR policies and activities, and instead show the agentic behaviours through which employees shape their work meaningfulness in the context of CSR. With this perspective, we contribute to the understanding of how organisations and individuals interact in the creation of meaningful work in the context of CSR.

Second, our study shows that employees can find meaningful work in organisations where CSR implementation is still emerging. This is important because only very few organisations ‘perfectly' implement (or embed) CSR in their day-to-day operations. At the same time, organisations should be aware that employees might find meaningful work through these meaning-infusing behaviours in the context of CSR, but that not all these behaviours are desirable for the organisation. Also, although our findings emphasise the proactive role of employees in the context of CSR, it does not mean that the enabling of meaningful work should be solely their task. Organisations should take the responsibility in continuing to embed CSR in it to provide context that supports employees' search for work meaningfulness.

Our third consideration for management practice, is related to the potential risks. Our findings show that some employees engage in rule-breaking behaviour to enhance their postitive impact on beneficiaries. Despite the prosocial intentions of this rule-breaking behaviour, it poses risks to the organisation, the resident and the employee. We found that when employees do not feel that there are opportunities to create structural change and ‘enact organisational structures', they ‘navigate' the existing structures, including rules and regulations.

## Data availability statement

The qualitative data presented in this article are not available because they contain information that could compromise the privacy of research participants and organizations involved in the study. Agreements were made to treat the data in an anonymous and confidential manner. Any further inquiries can be directed to the corresponding author/s.

## Ethics statement

Ethical review and approval were not required for the study on human participants in accordance with the local legislation and institutional requirements. Verbal informal consent was received prior to the data collection from all respondents.

## Author contributions

JJ: conceptualization, data analysis, writing, editing, and project administration. EL: conceptualization, writing, and editing. CW and SK: writing and editing. All authors contributed to the article and approved the submitted version.

## Conflict of interest

The authors declare that the research was conducted in the absence of any commercial or financial relationships that could be construed as a potential conflict of interest.

## Publisher's note

All claims expressed in this article are solely those of the authors and do not necessarily represent those of their affiliated organizations, or those of the publisher, the editors and the reviewers. Any product that may be evaluated in this article, or claim that may be made by its manufacturer, is not guaranteed or endorsed by the publisher.

## Footnotes

^1^It is an inductive study that explores employee's experiences and reactions to CSR initiatives in their pursuit of meaningful work. In this section, we provide an overview of the literature and concepts that we consulted either before or during the data analysis to inform and make sense of our emergent findings (Suddaby, 2006; Gioia et al., 2013).

^2^We have changed the names of the organisations to preserve anonymity.
